# CALENDAR

**DOI:** 10.1038/sj.bjc.6690260

**Published:** 1999-03


					1113 April 1999
MICRO 2000 International Microscopy Exhibition and
Conference
London, UK
Further information from:
Alison Winton, Exhibition Organizer, Royal Microscopical
Society, 37/38 St Clements, Oxford OX4 1AJ, UK.
E-mail: exhibitions@rms.org.uk
16 April 1999
Joint Meeting of the Section of Urology, Royal Society
of Medicine and the British Prostate Group - Prostate
Cancer: The Impact of Basic Science on Screening,
Treatment and Quality of Life
London, UK
Further information from:
Meeting Secretariat, c/o British Association of Urological
Surgeons, 35/43 Lincoln's Inn Fields, London WC2A 3PN, UK.
Tel: +44 (0) 171 405 1390, Fax: +44 (0) 171 404 5048
1719 May 1999
Radiology 1999-Imaging, Science & Oncology
International Convention Centre, Birmingham, UK
Proferred papers are invited for poster presentation. Deadline for
Work in Progress: 22 February 1999
Further information from:
Radiology 1999 Secretariat, 36 Portland Place, London W1N 4AT,
UK. Tel: +44 (0) 171 307 1410, Fax: +44 (0) 171 307 1414
CALENDAR
1632
British Journal of Cancer (1999) 79(9/10), 1632
(c) 1999 Cancer Research Campaign
Article no. bjoc.1998.0260

				

